# Overview of the radiographers’ practice in 65 healthcare centers using digital mammography systems in Portugal

**DOI:** 10.1007/s13244-017-0550-9

**Published:** 2017-03-16

**Authors:** Cláudia Sá dos Reis, Ana Pascoal, Lucian Radu, Mário Fartaria de Oliveira, João Alves

**Affiliations:** 1 Department of Medical Radiation Sciences, Curtin University, Perth, Western Australia; 2 Escola Superior de Tecnologia da Saúde de Lisboa/Instituto Politécnico de Lisboa (ESTeSL/IPL), Lisboa, Portugal; 3 Faculdade de Engenharia, Universidade Católica Portuguesa, Estrada Octávio Pato, 2635-631 Rio de Mouro, Portugal; 4 King’s College Hospital NHS Foundation Trust, Department of Medical Engineering and Physics, London, UK; 5 Department of Radiology, Centre Hospitalier Universitaire Vaudois (CHUV) and University of Lausanne (UNIL), Lausanne, Switzerland; 6 Instituto Superior Técnico (IST), Laboratório de Proteção e Segurança Radiológica (LPSR), Universidade de Lisboa (UL), Estrada Nacional 10 (ao km 139,7), 2986-066 Bobadela, Portugal; 7 Centro de Ciências e Tecnologias Nucleares (C2TN) do IST, UL, EN 10 (ao km 139,7), 2986-066 Bobadela, Portugal

**Keywords:** Radiographer, Digital mammography, Practice, Technique, Positioning

## Abstract

**Purpose:**

To assess current practices in digital mammography (DM) in Portuguese healthcare providers using digital systems. To investigate compliance with European standards regarding mean glandular dose and quality control practice and to identify optimisation needs.

**Methods:**

Two questionnaires, targeted at breast radiographers and chief radiographers, were designed and applied in 65 imaging departments offering DM. Questions fielded were focused on the staff profile and technical/clinical practice.

**Results:**

Prior to starting their activity in DM, 70% (82 out of 118) of the respondents received training in DM. The practice in 29 out of 59 providers was established by the manufacturers’ recommendations for image acquisition. Variations were observed between radiographers who belong to the same provider namely the selection of exposure parameters such as the target-filter combination and automatic mode. The use of the manual exposure mode was reported for imaging breast implants (44%) and surgical specimens (22%). The main causes of repeat examinations were skin folding (21%) and absence of pectoral muscle (PM) (20%).

**Conclusions:**

The study revealed opportunities to optimise radiographers’ practice in DM regarding the selection of exposure parameters. A robust and consistent training programme in DM and established local protocols can help to reduce the variations observed and improve clinical practice.

**Main Messages:**

*• Radiographers adopted different practices selecting AEC modes and T/F combinations*.

*• Radiographer practice is more consistent using DR than using CR systems*.

*• The main causes for rejecting images were the visibility of skin folding and PM absence*.

*• Radiographers were partly unaware of the dose indicator*.

*• Radiographers’ training needs: QC, interventional procedures and breast dose optimisation*.

## Introduction

Digital mammography (DM) is in use worldwide and is most commonly used in America, Europe, Australia and Japan. The International Cancer Screening Network (ICSN) cited in its 2008 report that 15 out of 27 European countries implemented DM in their breast screening programmes [1]. In the US, the Food and Drug Administration approved DM in 2000 but its adoption in screening mammography programmes has been slow. In 2006 less than 10% of mammography systems were digital [1]. In the UK, mammographic screening is coordinated by the National Health Service Breast Screening Programme (NHSBSP), which has implemented DM at their centres. As of July 2011, 85% of breast screening units had at least one DM set [2].

Obstacles to the introduction of DM include the high capital cost of equipment including archiving facilities, integration with existing X-ray systems, staff training and workflow reengineering [3, 4]. The successful transition from screen film to DM and the cost-effective use of DM require a clear understanding of the potential and limitations of DM systems as well as their potential impacts on the established routines [5–7]. Fully integrated DM systems offer opportunities to streamline workflow and increase workload [7–9]. Prior to clinical use of DM, specialised training for radiographers and radiologists is essential as well as continuous refresher training to update knowledge and promote competent and safe use of the technology. The establishment of clinical protocols and multidisciplinary meetings and feedback is important to audit practice and identify opportunities for optimisation. These strategies are essential to promote high-quality standards whilst minimising the radiation dose to the patient [7, 10–12].

Various organisations [e.g. the European Commission, International Atomic Energy Agency (IAEA), Institute of Physics and Engineering in Medicine (IPEM) and National Health Service Breast Screening Programme (NHSBSP)] release guidelines aimed at promoting quality in mammography and high-quality breast care. The currently available guidelines provide advice on organisational, technical and clinical matters in DM [13–15].

In Portugal, DM was introduced in clinical practice in 2000 and it is currently in use by various healthcare providers for screening, diagnosis, intervention and follow-up. No clinical audit data have been found to assess the implementation of DM in Portugal or the impact of arrangements put in place to promote cost-effective use of the imaging modality in the national health system. In fact studies combining all areas regarding radiographer practice in DM are scarce worldwide. Usually, the studies are limited in scope and focus only on specific subjects such as communication with patients, patient positioning, quality control practice, image evaluation and perception, breast compression or other breast imaging techniques [16–24].

The objectives of this study were to survey the academic and professional profile of radiography staff performing mammography and to characterise their routine practice in the use of the digital technology. It also aimed at providing recommendations to optimise the quality of DM performed in Portugal.

## Methods

### Development, testing and application of questionnaires

Two original questionnaires were designed to implement the survey. One was targeted to radiographers performing mammography and the other was targeted to chief radiographers who are frequently involved with the management of DM in Portuguese hospitals. Before sending them, nine radiographers piloted the questionnaires and suggestions were incorporated to improve the quality of the tool.

The questions were designed to capture data on the following themes: demographic profile such as age and gender, experience in radiography and DM, specialised education and training in DM and self-assessed training needs (Table 1).

**Table 1:**
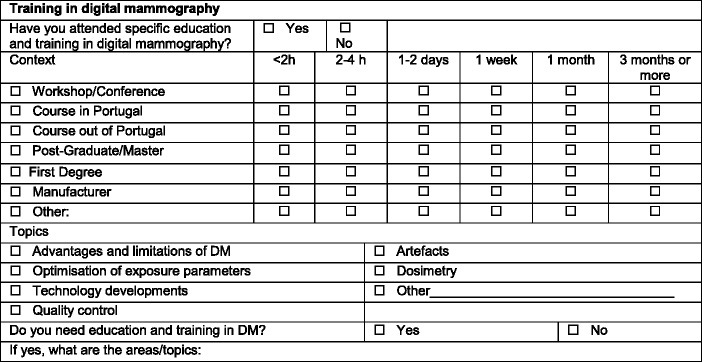
Summary of the questionnaires targeted to radiographers illustrating the questions to capture data regarding education and training in digital mammography

Data on the type of mammography system available at the facility, e.g., computed radiography (CR), direct digital mammography (DDM), most frequently used technique, e.g., manual vs. AEC, selected target-filter (T/F) combination, and the use of the breast dose (or exposure) indicator (Tables 2, 3 and 4), were also collected. Additionally, the variability in practice amongst radiographers working at the same centre regarding the protocol selected (T/F combination and AEC mode) was also investigated.

**Table 2:**
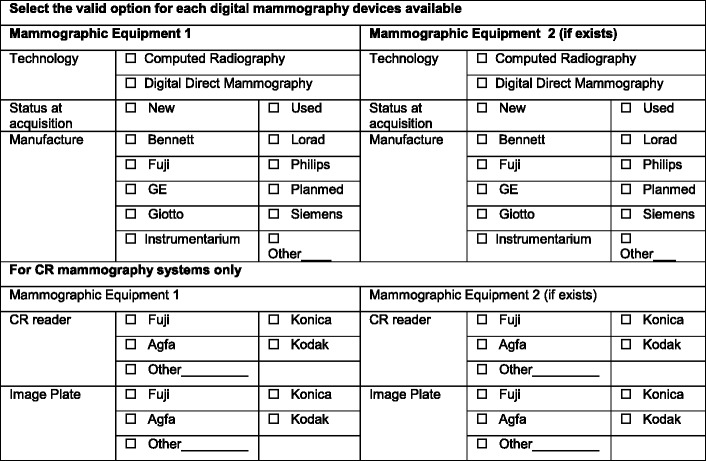
Summary of the questionnaire section to capture data on the specifications of the mammographic equipment available at the healthcare provider questioned (target: breast radiographer and chief radiographer)

**Table 3:**
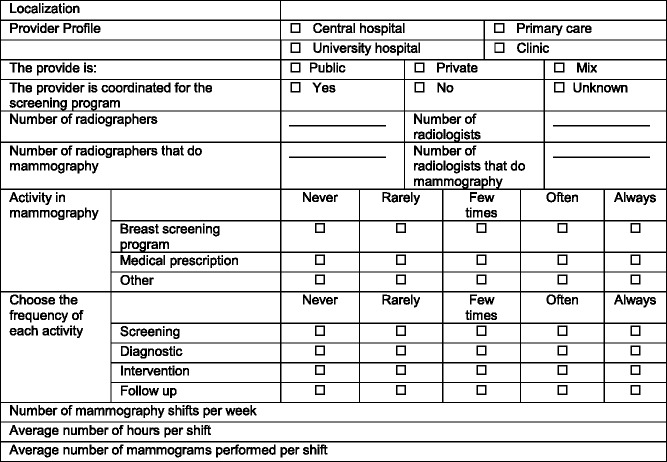
Summary of the questionnaire targeted to the chief radiographer: questions to capture data to characterise the type of healthcare provider, activity and workload

**Table 4:**
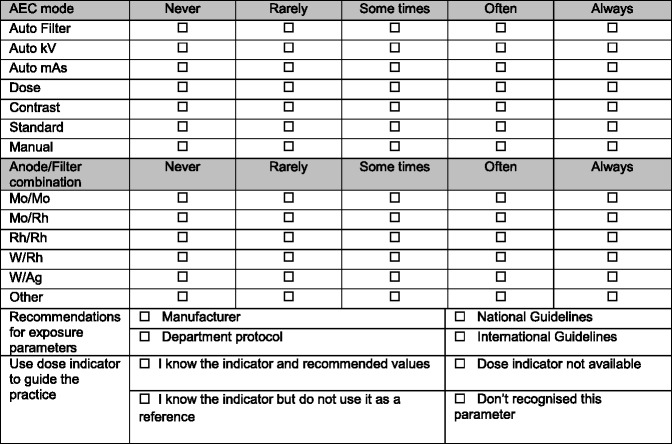
Summary of the questionnaire targeted to radiographers to capture data regarding the radiographic technique used and the reference guidance used to support usual practice

The questionnaire was also designed to capture information on the use of guidelines to support the practice in place. It collected data on staff preferences and views about the impact of DM on established practice, namely in radiographic technique, workflow and workload (Tables 3 and 4).

The questionnaires were posted or sent by e-mail to all providers of mammography services using DM technology (CR or DR) in continental Portugal and the Azores and Madeira Islands. The healthcare providers invited to participate in the study included large public hospitals, university hospitals, private hospitals and diagnostic clinics. The national coordination centre for breast screening was also invited to take part in the study.

A cover letter informing about the context of the study and its objectives accompanied the questionnaires. Contacts were established with the hospital administration and with the local radiology department. The opportunity to take part in an independent assessment of compliance with the best practice in mammography was highlighted as a benefit to the healthcare provider.

The data collected were screened for quality and the descriptive statistical analysis was performed using the software packages MS Excel (version 2007, Microsoft ©) and SPSS (version 19, IBM).

## Results and Discussion

The questionnaires were sent out to 270 institutions and 118 responses were received from 65 centres representing a response rate of 24.1%.

### Radiographers’ profiles

#### Age and gender

The majority of radiographers (98%; n = 118) were female within the age range 20 to 59 years old. Among these were a significant number of young professionals (46%) aged 20–29 years. Since mammography is a diagnostic modality used most commonly for imaging female breasts, it seems natural that the healthcare staff should be mainly composed of women as this may also contribute to the patient’s acceptance and self-assurance. Published studies [25, 26] indicate that some women undergoing mammography may feel embarrassed when assisted by a male radiographer. Fitzpatrick et al. [25] reported that overall, 17.5% of women agreed (or strongly agreed) with the statement “If there were male radiographers I would not return to BreastCheck (Irish screening program) for another screening appointment and a further 18.3% were unsure”.

#### Specific education and training in digital mammography

Many responding breast radiographers (88%; 96/118) had graduated in radiography. The average work experience in radiography was 10 years ranging between 1–39 years. The average work experience in DM was 4.7 years (range: 1–15 years).

Prior to enrolling as DM radiographers, the majority (70%; n = 118) of participating radiographers had received training in DM. A few (3%) (4) received training by attending courses on general radiology techniques. Participants stated that they received training from the manufacturer’s study days (50), through workshops (39) and/or other training courses (21). The reported duration of the training varied with a predominance of short-term (1–2 days) sessions (73%). Longer training periods (≥1 month) were less frequent (27%). Topics covered in the training included advantages and limitations of digital mammography, optimisation of exposure parameters, technological developments in mammography and artefacts recognition.

More than one third of the respondents (38%) reported self-assessed the need to refresh their knowledge on DM. Key areas of training needs that were highlighted were on quality control (QC), interventional procedures and breast dose optimisation. A previous study [10] about training on medical imaging concluded that when compared to CT and MRI, mammography was a less valued imaging modality, which has also been highlighted in other European studies [11, 27]. As per current practice, qualified radiographers graduating in Portugal are entitled to start performing DM promptly following graduation. Anecdotal evidence suggests that experienced staff usually informally supervise radiographers starting to practice mammography. Currently there is no established complementary training programme in place nationwide or a formal continuous professional development (CPD) training of 40 h/year as recommended by the EUREF guidelines.

### Usual radiographic practice with digital mammography systems

#### Support guidance for the technical protocol in use

**Fig. 1:**
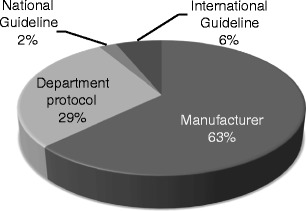
Guidance to support mammography practice in the participating centres

The majority (63%) of centres followed the recommendations provided by the manufacturer to select exposure parameters on the DM systems. Some centres (29%) reported using protocols developed locally and a small percentage (6%) stated using international guidelines [American College of Radiology (ACR), European Protocol (EUREF)] to support quality assurance in mammography (Fig. 1).

#### Exposure mode (AEC vs. manual)

**Fig. 2:**
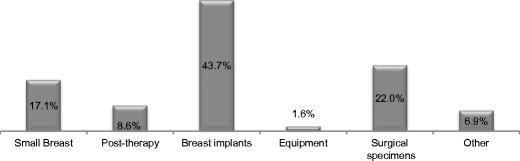
Justification for the use of manual exposure mode in mammography

Mammography imaging units incorporate an Automatic Exposure Control (AEC) system. This provides a means of achieving adequate and consistent image quality (IQ), independent of the breast characteristics and the radiographer’s experience in the selection of the X-ray tube exposure parameters [28–30]. AEC systems can operate in various modes that are manufacturer-specific. For each system usually three modes are provided offering a range of IQ options (lower, standard and higher) selected by the radiographer according to the clinical task (diagnostic or screening). The use of the manual mode or manual selection of the settings by the operator is not recommended for standard mammography with few justified exceptions, like in the cases of breast implants, surgical specimens and breasts that have undergone surgery or radiotherapy [31–33]. This was observed in our sample: manual mode selection was mainly used when imaging breast implants (44%), surgical specimens (22%) collected during biopsies, small breasts and post-radiotherapy breasts (Fig. 2).

#### Target-filter combination

**Fig. 3:**
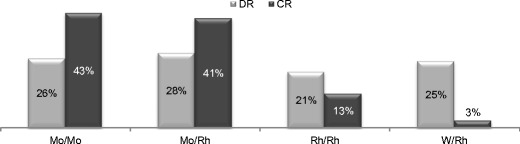
Frequency distribution of target-filter combinations used in mammographic examinations with the CR and DR system (AEC)

The target/filter (T/F) combinations available were Mo/Mo, Mo/Rh, Rh/Rh and W/Rh (Fig. 3). For CR systems Mo/Mo and Mo/Rh were the most frequently used, Rh/Rh was reported in 13% of the cases and W/Rh was rarely used. For DR systems Mo/Rh seemed to be the most frequent T/F combination but Mo/Mo, Rh/Rh and W/Rh were also frequently used.

#### Inter-radiographer variability (use of technical protocol)

**Fig. 4:**
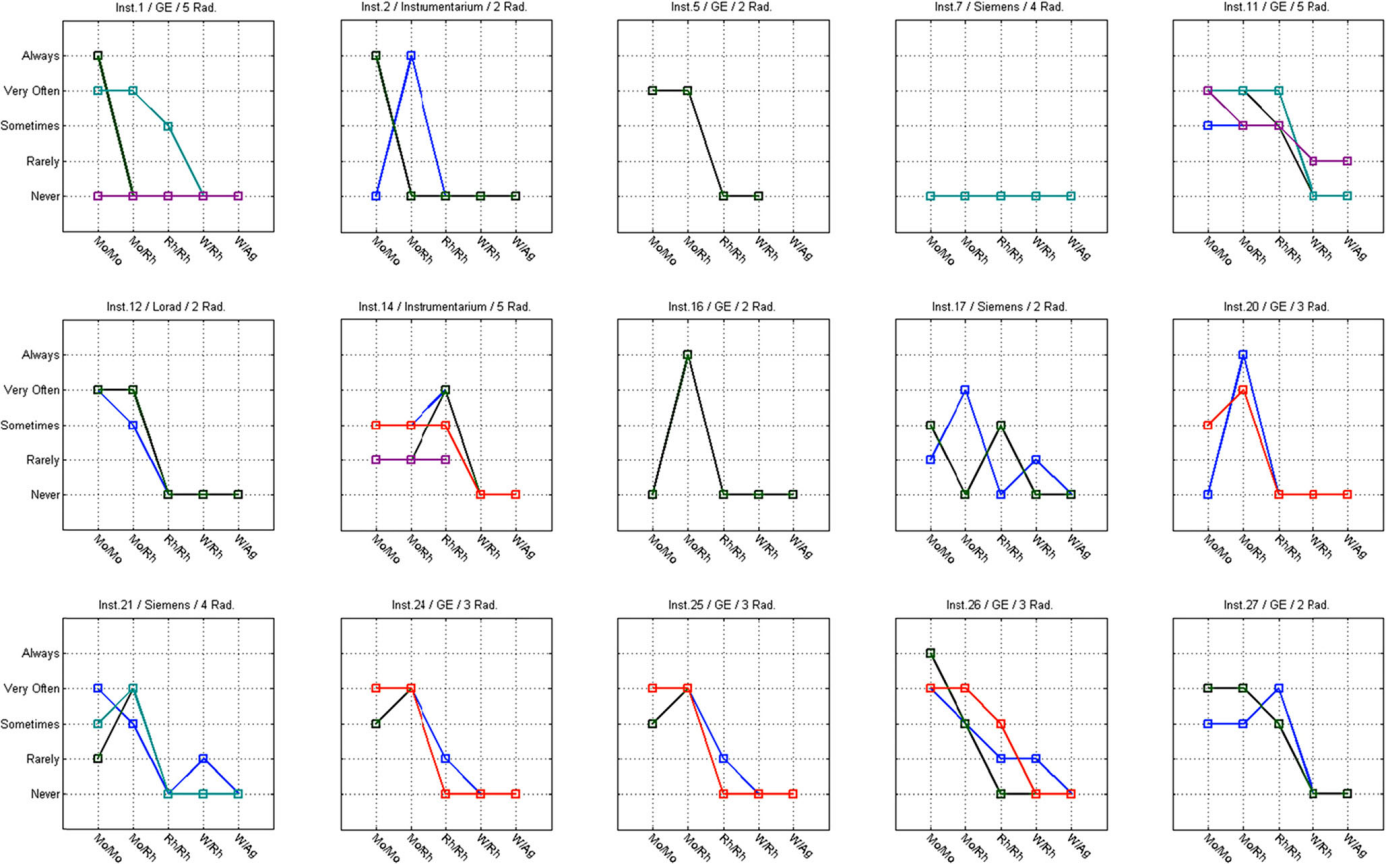
Variations in the technical protocol [target-filter (T/F) combination] amongst individual radiographers employed at the same centre used to produce mammography images with CR systems

**Fig. 5:**
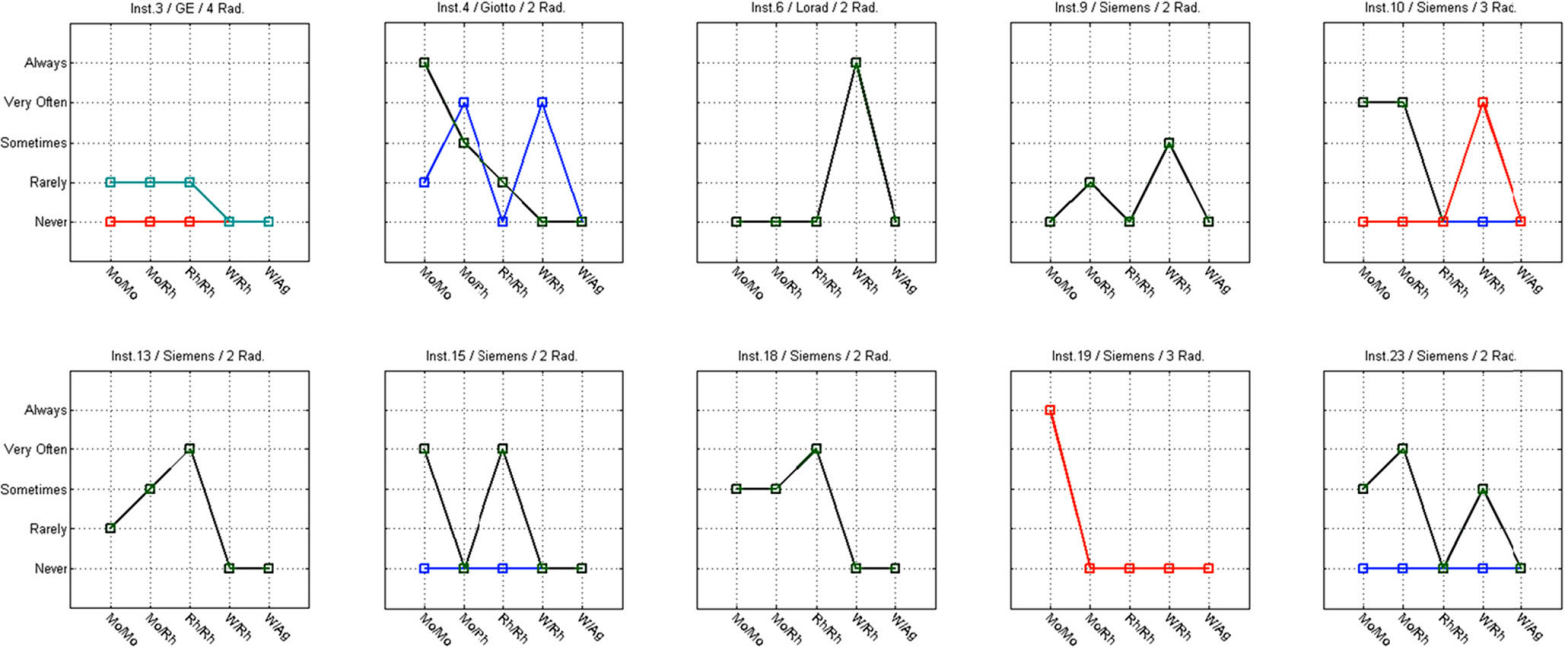
Variations in the technical protocol [target-filter (T/F) combination] amongst individual radiographers employed at the same centre used to produce mammography images with DR systems

**Fig. 6:**
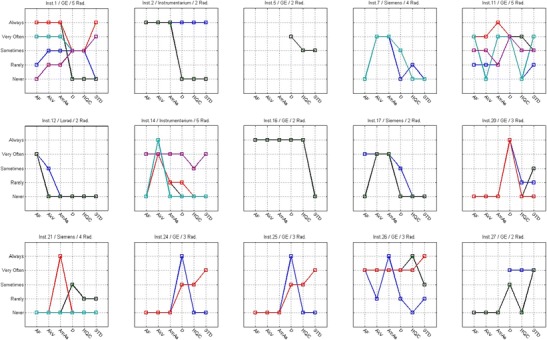
Variability in the use of AEC mode in mammography (CR system) for various members of staff (radiographers) and various institutions (inst.) and manufacturers

**Fig. 7:**
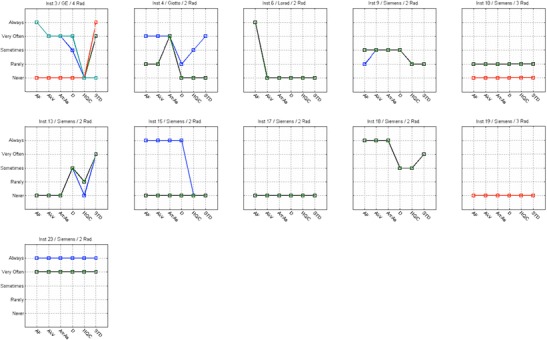
Variability in the use of AEC mode in mammography (DR system) for various members of staff (radiographers) and various institutions (inst.) and manufacturers

The analysis of Figs. 4, 5, 6 and 7 shows that in some centres some radiographers adopted different practices in the selection of T/F compared to their colleagues. This was observed for CR systems at centres 1, 2, 11, 14, 17, 21, 26 and 27 (Fig. 4). The radiographers from institution 11 (Fig. 4) reported using the W/Ag combination, which was not available on the mammography equipment they were using. This may represent a lack of clarity from staff regarding the feature of the equipment in use.

For DR systems the overall practice adopted radiographers was more consistent. However, variations in the selection of T/F were noticed for centres 4, 10, 15 and 23.

Regarding the selection of AEC mode, a spread of scores was observed for both CR and DR systems showing variations between the centres (Figs. 6 and 7). The practice of individual radiographers appears to be consistent. A higher variability was shown for CR namely in institutions 1, 2, 7, 11, 14, 21, 26 and 27. The available AEC modes in mammography devices are dependent of each manufacturer, and according to radiographers they followed the manufacturer recommendations to choose the exposure mode. However, the answers were not consistent with the options that are available in the equipment for the majority (75%) of the institutions that were considered. Radiographers’ training in DM is expected to have a significant impact on their practice. A clear understanding of the differences between the AEC modes provided by the equipment (as well as the rationale for selection) is important to select the technical protocol in mammography.

As discussed above, 33% of radiographers have not had specific education and training in mammography and reported little experience in using the technology. Also, the use of local protocols and guidelines for good practice was not consistently adopted. These factors could contribute to the observed variations in practice amongst radiographers in the selection of exposure settings.

#### Analysis of rejected/repeated examinations

**Fig. 8:**
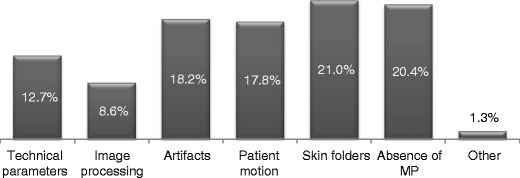
Causes for rejection and repeat of mammography images resported by breast radiographers

About 88% of radiographers stated that they rarely needed to reject and/or repeat mammography examinations. When examinations needed to be rejected and repeated, they reported that the main causes for this were the visibility of skin folding on the image (21%), absence of pectoral muscle (PM) (20%), blur caused by patient motion (18%) and/or presence of other types of artefacts in the image (18%) (Fig. 8). Less frequent causes for rejection were inappropriate image processing and/or technical parameter selection. However, the majority (65%) of radiographers do not perform analysis of rejected/repeated mammography examinations in a systematic way as recommended by European guidelines (and ACR guidelines) affecting the opportunity to identify opportunities for optimisation.

Assessment of rejected/repeated images to identify the causes of rejection is a valuable quality assurance practice also recommended by ACR and IAEA guidelines [14, 34–36]. Implementing corrective and preventive measures to reduce the number of repeated mammography examinations is important to ensure that mammographic images are produced at high quality/standards [14, 35] and comply with the ALARA/ALARP principles. Additionally, financial gains are expected because of the most efficient use of the equipment and radiographers’ time.

#### Use of the dose (or exposure) indicator

Monitoring and optimising the dose to the patient in mammography is a recommended quality control procedure by all international guidelines to ensure that the risk to the patient is kept low.

For DR systems the mean glandular dose (MGD) to the breast can be monitored promptly following the exposure using the dose indicator incorporated in the majority of DM systems. For mammography with CR systems an MGD value is usually not promptly available to the user. An indication of exposure on the image receptor is used as it has an effect on patient dose (and also on image quality). The name of the index varies depending on the manufacturer. The responses to the questionnaire showed that half (50%) of the radiographers were aware of the existence of a dose indicator on the equipment and used its displayed value to monitor the dose to the patient following the mammography procedure. About 11% referred to being aware of the indicator but not using it. A substantial percentage (21%) of the respondents were not aware of the indicator’s existence at all.

The analysis of the exposure indicator system is an important QC practice that alerts the radiographer to the occurrence of sub-optimal exposures; these may have a direct impact on the IQ and patient dose. Considering the results of the survey it may be appropriate to provide radiographers with refresher training about the dose in mammography. The training should highlight the QA tools provided by the equipment such as the dose indicator. Additionally, mechanisms to assess the impact of training, such as auditing, should be put in place. Appropriate communication and feedback mechanisms among all staff members involved in mammography will also be important to promote consistent practice.

### Personnel views on the impact of digital mammography

**Fig. 9:**
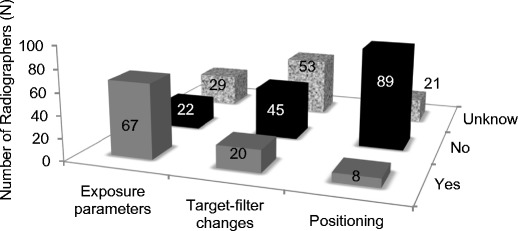
Areas where changes were observed following the introduction of digital technologies for mammography (radiographers’ personal view)

More than half (57%) of the total number of radiographers reported having noticed changes in practice following the introduction of DM, particularly changes in the exposure factors used. Their perception was that digital mammography decreased the patient dose compared to screen-film mammography because of the reduction of exposure time and increase of beam energy. They also reported that the introduction of AEC modes caused reduction of the number of repeated images. Variations in the T/F combination in use were reported by 17%, mainly referring to a decrease in use of Mo/Mo and increased use of W/Rh (Fig. 9).

The majority of radiographers (75%) considered that the introduction of DM did not require changes to the positioning of the patient. A few radiographers (7%) reported that positioning small breasts is now more difficult compared to analogue systems because of the larger platform size. Other authors also reported challenges with positioning of small breasts on large platforms and increased challenges to fully fulfil the recommended criteria of good radiographic positioning practice [13, 35, 37]. When positioning small breasts there is a risk of including part of the arm in the image and sometimes also part of the abdominal wall, which is not desirable.

**Fig. 10:**
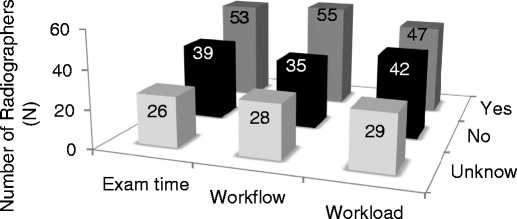
Impact of digital mammography on workload, workflow and examination time (radiographers’ point of view)

Approximately half of the radiographers considered that the introduction of DM caused impacts on workload, workflow and examination time (Fig. 10). Image acquisition was faster and the number of mammography procedures performed per shift had increased. It was reported that time between consecutive examinations also increased allowing radiographers to dedicate more attention to patients.

## Conclusions

This study collected evidence and provided an overview of radiographers’ profile and practices in use in digital mammography in Portugal.

The majority of radiographers were young females with little experience in DM. Specialised training in mammography is not mandatory and radiographers were trained on the job and worked under supervision. The majority of radiographers identified self-assessed need for training on digital mammography with focus on artefact recognition, dosimetry and quality control. Limited evidence of compliance with the recommended international standards of good for mammography practice (EUREF) was found as the radiographers do not perform 40 h/year of CPD in mammography and quality control tests are not performed following the main recommendations provided by the EUREF guidelines.

The study revealed opportunities for optimisation of radiographer practice in digital mammography in Portugal. A robust and consistent training programme in digital mammography for radiography staff can help reduce the observed variations in practice. The training should take into consideration the activities of the radiographer and include practice with the equipment. It should be developed by a multidisciplinary team with the input of the relevant stakeholders (radiographers, radiologists and medical physicists). The establishment of an international training network for mammography is likely to provide a valuable contribution to improve and disseminate best practice in digital mammography.
